# The Impact of Saline Water on Women’s Health in the Coastal Region of Bangladesh: Special Attention on Menstrual Hygiene Practices

**DOI:** 10.7759/cureus.67032

**Published:** 2024-08-16

**Authors:** Susmita Sinha, Rahnuma Ahmad, Kona Chowdhury, Farhana Ferdaus, Suman Banik, Miral Mehta, Santosh Kumar, Md. Ahsanul Haq, Mainul Haque

**Affiliations:** 1 Department of Physiology, Enam Medical College and Hospital, Dhaka, BGD; 2 Department of Physiology, Medical College for Women and Hospital, Dhaka, BGD; 3 Department of Pediatrics, Enam Medical College and Hospital, Dhaka, BGD; 4 Department of Community Medicine, Khulna City Medical College and Hospital, Khulna, BGD; 5 Department of Administration, Directorate General of Health Services, Ministry of Health and Family Welfare, Dhaka, BGD; 6 Department of Pedodontics and Preventive Dentistry, Karnavati University, Gandhinagar, IND; 7 Department of Periodontology and Implantology, Karnavati School of Dentistry, Karnavati University, Gandhinagar, IND; 8 Department of Biostatistics, International Centre for Diarrhoeal Disease Research, Bangladesh (icddr,b), Dhaka, BGD; 9 Department of Research, Karnavati Scientific Research Center (KSRC), Karnavati School of Dentistry, Karnavati University, Gandhinagar, IND; 10 Department of Pharmacology and Therapeutics, National Defence University of Malaysia, Kuala Lumpur, MYS

**Keywords:** reproductive health issues, salt water, koyra, lack of fresh water, socioeconomic status, menstrual hygiene, women, health, coastline, salinity

## Abstract

Introduction

Salinity intrusion is the most common global concern along coastlines, but it can happen inland also. The lack of freshwater is the primary issue affecting the coastal areas. Many health problems are prevalent among the inhabitants due to their frequent use of salted water. The health of women living along the coastline is getting progressively compromised due to salinity intrusion.

Objectives

The study aims to determine menstrual health practices and other health problems faced by women in the coastal region of Bangladesh.

Methods

The study was conducted using a survey research design from May 2023 to October 2023 on rural women aged 18 to 45 years, who lived in the Bangladeshi village of Koyra Upazila in the Khulna district. The sample size of the study was 101. Open- and closed-ended questions from a planned interview schedule were used to gather primary data. Additional information from appropriate sources, e.g., newspapers, publications, and books, was utilized to enhance the comprehensiveness of statistical analysis and support rationality. A p-value of 0.05 was considered significant. Statistical analysis was performed using STATA version 15 (StataCorp LLC, College Station, TX).

Results

Menstrual hygiene practices showed a higher prevalence of using fabric rags and reusing them after rinsing them in salt water (72.3%) than sanitary pads (25.7%). It was observed that the risk of diarrhea among tubewell water and rainwater users was significantly lowered by 0.25 times (95% CI = 0.06, 0.99; p = 0.049) and 0.06 times (95% CI = 0.01, 0.43; p = 0.005), respectively, compared to pond water users.

Conclusion

Salinity has a significant impact on the livelihoods and health of coastal women. The village women are unaware of the health risks of excessive saline water use. Establishing an adequate supply of freshwater reservoirs for the entire community throughout the year is an alternative for women to use as a source of water for hygiene necessities.

## Introduction

Salinity intrusion is a global issue that is most prevalent at coasts, and this compromises the sustainability of freshwater provision and impacts the ecosystem, soil, and water quality [1]. The increase in seawater levels has particularly impacted Bangladesh, which is among the region's most susceptible to climate change. Surface water, groundwater, and minerals in the soil have become salinized as a result of increased saltwater infiltration in low-lying coastal regions, lagoons, and river channels, as stated in a report by the Intergovernmental Panel on Climate Change (IPCC) [2]. In the southwest coastal region of Bangladesh, saltwater invasion is causing severe health issues. As a result of seawater permeation and coastal tide flooding, soil with salt content is primarily found in coastal districts. Since seawater invasion raises the salt level of surface water, it harms individuals who live alongside the coast [3].

The coastal population is affected by the changing climate in many aspects. Women in coastal communities face economic challenges and significant health repercussions. In addition, access to safe drinking water and menstrual hygiene might be challenging for women and girls in this area [4,5]. Low-income households are regularly unable to purchase feminine hygiene products. Therefore, women and teenage girls frequently rely on cotton cloths that they rinse in continually salinized water. According to a study, women and girls in the vicinity are susceptible to various hygiene-related health problems, including dermatological conditions and reproductive health issues, since they can only rinse their clothes in dirty salt water and reuse them when they menstruate [6-8].

Climate change-induced increases in groundwater and surface water salinity have been found to constitute a significant health hazard for coastal residents in Bangladesh [9]. However, little is known about how salinity impacts the risk of water-related diseases and what can be done to reduce the environmental threat. This study focused on the effects of salinity on the health of coastal region's women, specifically in the Koyra Upazila of Khulna district in Bangladesh.

Problem statement

Bangladesh has a very high risk of natural disasters due to its location. Approximately 29% of people who reside on Bangladesh's coastline are more exposed to calamities resulting from cyclones, saltwater, floods, river erosion, and rains than in other parts of the world [10]. Besides, the southern part of the country is only two meters above sea level. In addition, 19 coastal districts have moderate to severe salinity levels, which have risen in the past several years due to the adverse effects of weather events on a small area [11]. Southwest Bangladesh is severely lacking in freshwater as a result of changing climates. Salinity is a significant problem for coastal areas because it impacts the soil and water essential to human health. Salinity in the water is linked to several health problems, including skin conditions, dysentery, diarrhea, and hypertension [12-14]. Women in coastal communities face more significant health challenges due to salinity. When girls and women use this water during their menstruation, pregnancy, and postnatal phases, they become more vulnerable to reproductive health issues. Female reproductive health issues are prevalent in areas with a lot of saline water. Furthermore, consuming saline water continuously raises the risk of prenatal hypertension and preeclampsia [15]. Since the scenario is becoming worse by the day, this problem must be appropriately addressed to provide efficient healthcare services for girls and women living in coastal areas as well as to bring about specific permanent resolutions.

Objectives of the study

This study aimed to observe the menstrual hygiene practices and other health issues that coastal women commonly endure due to climate change and increased saltwater intrusion. Additionally, this research appraised the risk of infection and water-borne diseases among various sources of water utilization.

## Materials and methods

**Figure 1:**
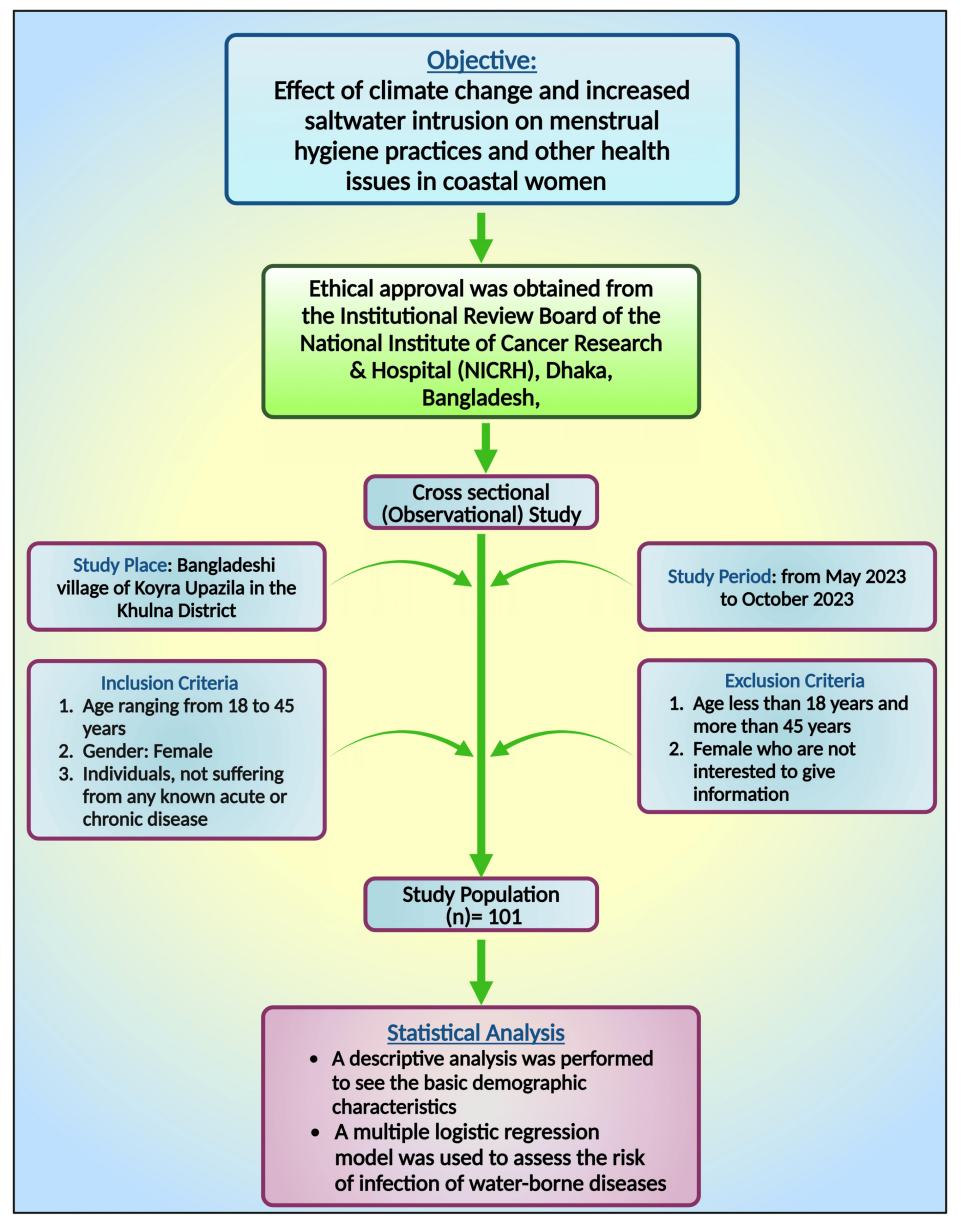
Flowchart showing the methodology of this study. Note: This figure was drawn using the premium version of BioRender (https://biorender.com/) [16], with the agreement license number FS272OMIID. Image credit: Susmita Sinha.

The villages of Koyra Upazila were purposefully chosen to fulfill particular study objectives and address the effect of salinity and water contamination on the health of rural women in Bangladesh. The study was conducted using a survey research design on rural women aged 18 to 45 years who lived in the Bangladeshi village of Koyra Upazila in the Khulna district from May 2023 to October 2023 (Figure 1). Open and closed-ended questions from a planned interview schedule were used to gather primary data. Besides, additional information from appropriate sources, such as newspapers, publications, and books, was utilized to enhance the comprehensiveness of statistical analysis and support rationality.

Study design

This was a cross-sectional, observational study conducted from May 2023 to October 2023. The study was conducted in Koyra, a coastal village in the Khulna district of Bangladesh. The total number of study subjects was 101 women who resided in Koyra Upazilla, Khulna, Bangladesh. The participant selection criteria are depicted in Table 1.

**Table 1:** Selection criteria for this research. * In Bangladesh, people under 18 years old are considered minors. Therefore, we cannot obtain written informed consent from them. Additionally, those aged 18 to 45 years old are considered to be of reproductive age. Therefore, this group matches our study objectives.

Inclusion criteria	Exclusion criteria
Gender: Female	Age under 18 years and more than 45 years*
Age ranging from 18 to 45 years	Subjects who have disagreed to participate
Any ill subject	

Sample size

The proposed study's required sample size has been calculated using the following formula: n = z^2^pq/d^2^. Here, n = sample size; z = the value of the standard variant at a given confidence level of 1.96; p = what we are trying to estimate (prevalence), and for the most conservative value, it is 0.5; d = 0.05; q = 1 - p, that is when p = 0.5; q = 1 - 0.5 = 0.5. Purposive sampling was used as the sampling technique.

Data collection

The data were collected through a structured questionnaire administered to the subjects who met the inclusion criteria. A face-to-face conversation was employed to collect the information. We can conduct medical and laboratory examinations. As this research was not funded, we cannot bear the financial overhead.

Ethical approval

This research obtained ethical approval from the Institutional Review Board of the National Institute of Cancer Research & Hospital (NICRH), Dhaka, Bangladesh, with reference number NICRH/IRB/2023/18 (dated: 15/03/2023). In addition, the participants received a thorough explanation from the researchers regarding their future publication plans and study objectives.

Statistical analysis plan

A descriptive analysis was performed to see the basic demographic characteristics. To assess the risk of infection of water-borne diseases among tubewell and rainwater users compared to pond water users, a multiple logistic regression model was used to estimate the p-value, and the model was adjusted by age, marital status, religion, and education. The considered level of significance was a p-value of 0.05. A multiple logistic regression model was used to estimate the p-value, and the model was adjusted based on age, marital status, religion, and education. The statistical analysis was performed utilizing STATA version 15 (StataCorp LLC, College Station, TX).

## Results

The summary of the demographic characteristics of 101 study participants is depicted in Table 2, showing a mean age of 40.9 years with a standard deviation (SD) of 5.23. Marital status revealed that the majority were married (72.3%, 73), followed by divorced (19.8%, 20), unmarried (3.0%, 3), and widowed (5.0%, 5). Religious beliefs: 72.2% (73) and 22.8% (23) of the study participants bear Islam and Hinduism, respectively. Education status indicates that 47.5% (48), 35.6% (36), and 16.8% (17) were illiterate, had primary education, and had secondary education, respectively. Occupations of the study participants vary, with homemakers comprising the largest group (41.6%, 42), followed by day laborers and cattle rearers (22.8%, 23) and school teachers (10.9%, 11). The family status reflected a predominance of the lower class (51.5%, 52) and the lower middle class (40.5%, 41). Housing predominantly comprised 65.3% (66) of katcha houses (mud houses) and 34.7% (35) of tin shed houses. Most women used pit latrines with no water seal (88.1%, 89) and tubewell for drinking water (59.4%, 60). Additionally, 59.4% (60) reported no water-borne diseases in the last three months, with diarrhea (27.7%, 28) and skin itching (12.9%, 12) prevalent among study participants. Regarding urinary symptoms, 55.4% (56) reported no symptoms of UTI, while 44.6% (45) reported lower abdominal pain (LAP) with burning micturition. Menstrual hygiene practices show a higher prevalence of using old cloth rags and reusing them after washing them in salt water (72.3%, 73) and sanitary pads (25.7%, 26).

**Table 2:** Demographic characteristics of the study participants. Data are presented as mean ± SD or number with percentage in parenthesis. LAP: lower abdominal pain; N/A: not available.

Variables		Observations (n = 101)
Age		40.9 ± 5.23
Marital status	Married	73 (72.3%)
	Unmarried	3 (3.0%)
	Divorced	20 (19.8%)
	Widow	5 (5.0%)
Religion	Islam	73 (72.2%)
	Hindu	23 (22.8%)
Education status	Illiterate	48 (47.5%)
	Primary	36 (35.6%)
	Secondary	17 (16.8%)
Occupation	Student	2 (2.0%)
	Day laborer	23 (22.8%)
	Cattle rarer	23 (22.8%)
	Housewife	42 (41.6%)
	School teacher	11 (10.9%)
Family status	Lower class	52 (51.5%)
	Lower middle class	41 (40.5%)
	Middle class	8 (7.90%)
Housing status	Katcha (mud house)	66 (65.3%)
	Tin shed house	35 (34.7%)
Latrine types	Pit latrine with water seal	12 (11.9%)
	Pit latrine without water seal	89 (88.1%)
Drinking water source	Tubewell	60 (59.4%)
	Rainwater	23 (22.8%)
	Pond water	18 (17.8%)
Water-borne diseases in the last three months	Not affected	60 (59.4%)
	Diarrhea	28 (27.7%)
	Skin itching	12 (12.9%)
Urinary symptom	No symptoms of UTI	56 (55.4%)
	LAP with burning micturition	45 (44.6%)
Menstrual hygiene	Use old cloth rags and reuse them after washing them in salt water	73 (72.3%)
	Use sanitary pad	26 (25.7%)
	N/A	2 (2.0%)

**Figure 2:**
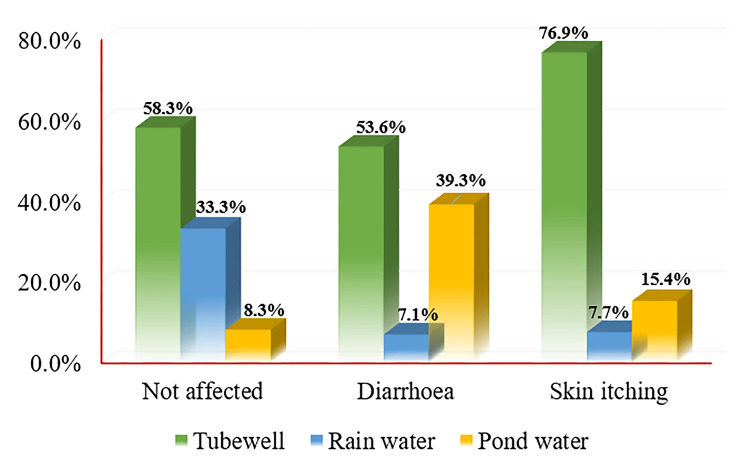
Distribution of water-borne diseases among users of different water sources.

Figure 2 presents the occurrence of water-borne diseases in the last three months among participants, categorized by their drinking water source (tubewell, rainwater, and pond water). Among participants who used tubewell as their drinking water source, 58.3% (35) reported not being affected, 53.6% (15) reported diarrhea, and 76.9% (10) reported skin itching (within the water-borne disease category). Of those study participants using rainwater, 33.3% (20) of participants reported not being affected, 7.1% (2) reported diarrhea, and 7.7% (1) reported skin itching. Among participants who used pond water, 8.30% (5) reported not being affected, 39.3% (11) reported diarrhea, and 15.4% (2) reported skin itching.

It was observed that the risk of diarrhea among tubewell water and rainwater users was significantly lowered by 0.25 times (95% CI = 0.06, 0.99; p = 0.049) and 0.06 times (95% CI = 0.01, 0.43; p = 0.005), respectively, compared to pond water users (Table 3).

**Table 3:** Risk of infection of water-borne diseases among tubewell and rainwater users compared to pond water users. Note: A multiple logistic regression model was used to estimate the p-value, and the model was adjusted based on age, marital status, religion, and education.

Variables	Odds ratio (OR)	p-value
Not infected	1 (Ref.)	
Diarrhea		
Pond water	1 (Ref.)	
Tubewell water	0.25 (0.06, 0.99)	0.049
Rainwater	0.06 (0.01, 0.43)	0.005
Skin itching		
Pond water	1 (Ref.)	
Tubewell water	1.14 (0.16, 8.07)	0.893
Rainwater	0.25 (0.02, 3.75)	0.317

**Figure 3:**
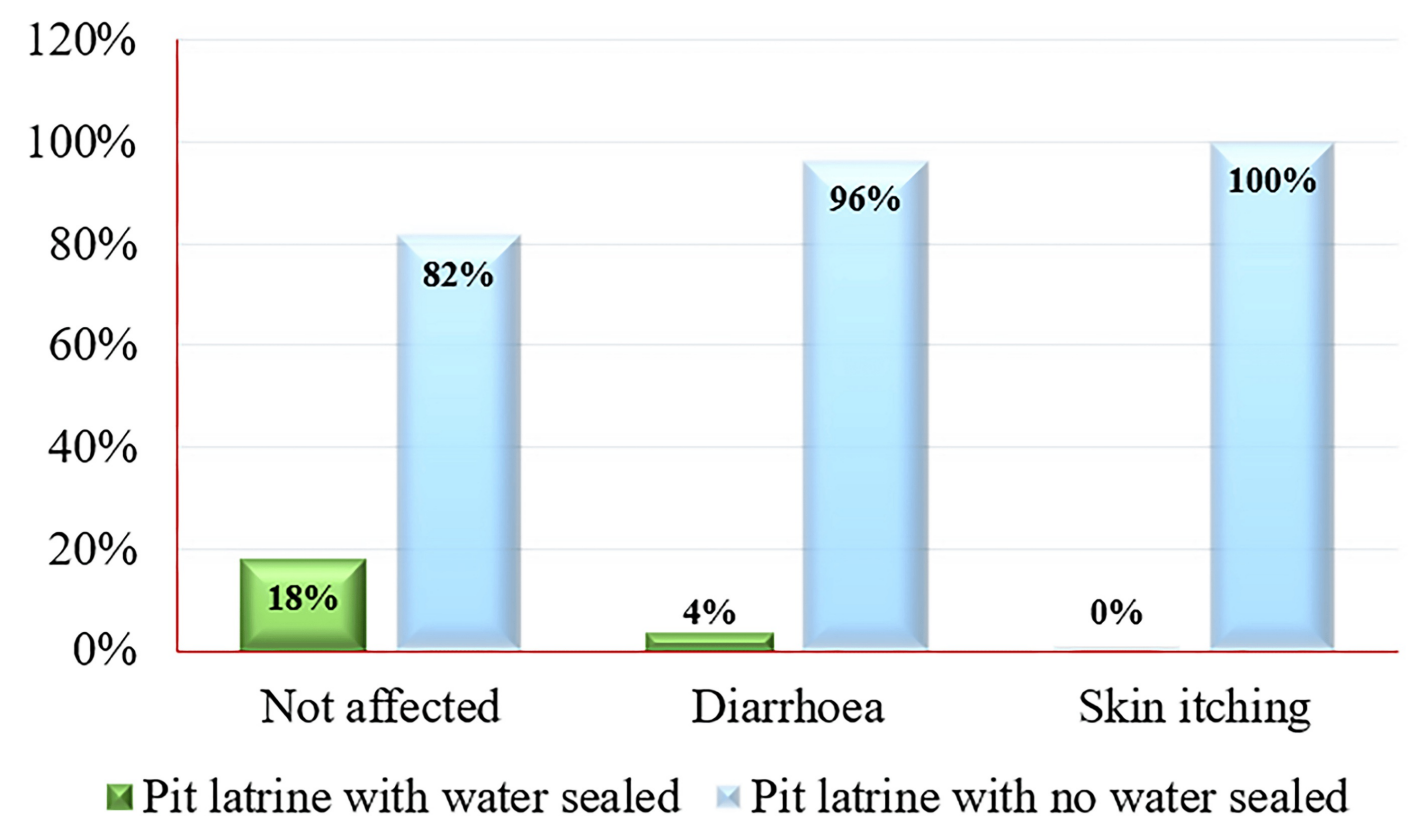
Distribution of water-borne diseases among users of different latrine types.

Figure 3 presents the occurrence of water-borne diseases in the last three months among participants, categorized by the type of lavatory used. Participants using pit latrines with water seal reported 18.3% (11) cases of not being affected, 3.6% (1) cases of diarrhea, and no cases of skin itching (within the water-borne disease category). Conversely, those using pit latrines without water seals reported 81.7% (49) cases of not being affected, 96.4% (27) cases of diarrhea, and 100.0% (13) cases of skin itching (within the water-borne disease category). Among 101 study participants, 59.41% (60) reported being unaffected by water-borne diseases, 27.72% (28) reported diarrhea, and 12.87% (13) reported skin itching. This breakdown provides insights into the relationship between the type of restroom used and the prevalence of water-borne diseases among the study participants.

**Figure 4:**
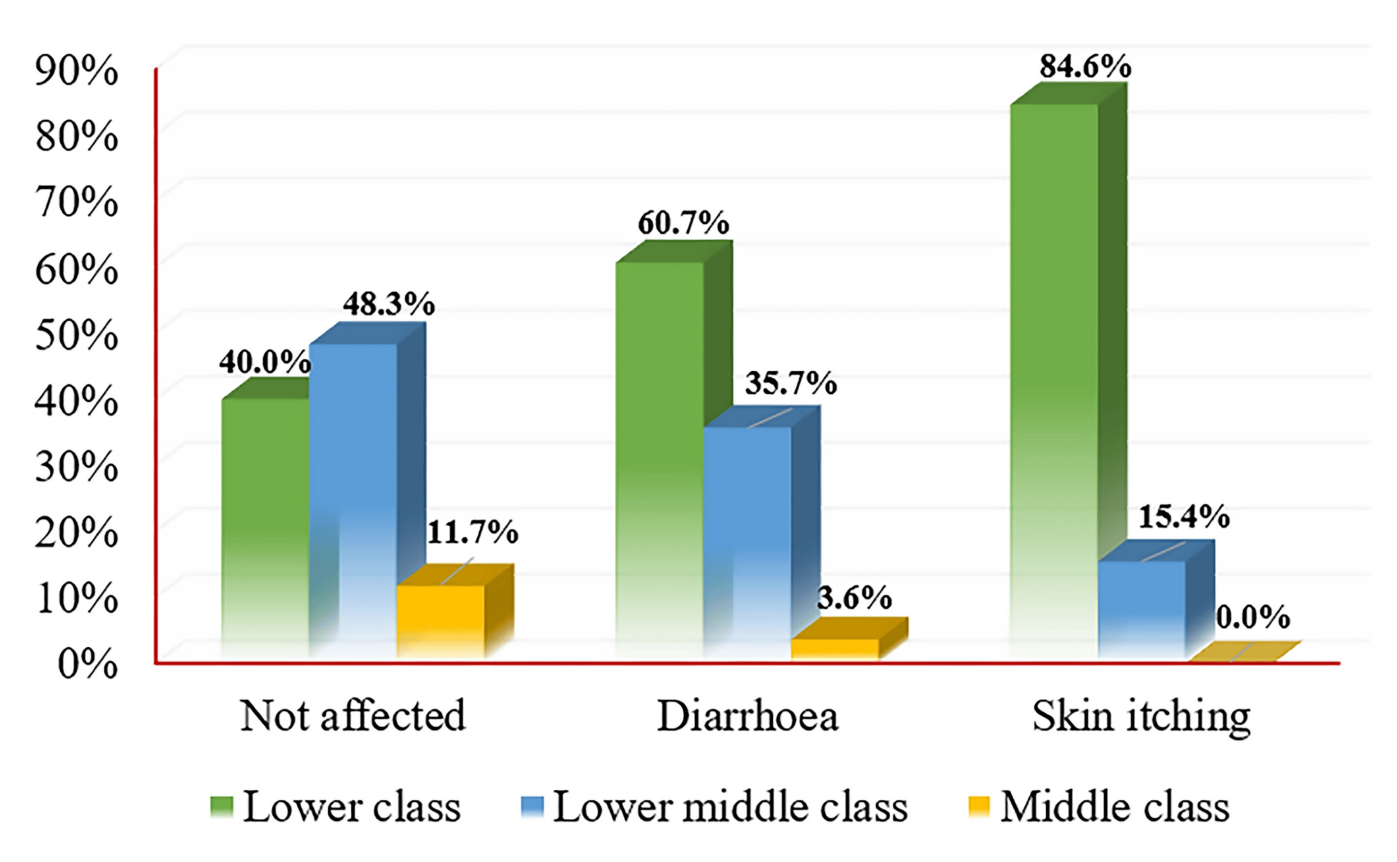
Distribution of water-borne diseases among participants of different socio-demographic classes.

Figure 4 presents the occurrence of water-borne diseases in the last three months among participants, categorized by their family status (lower class, lower middle class, and middle class). Among participants classified as lower class, 40.0% (24) reported not being affected, 60.7% (17) reported diarrhea, and 84.6% (11) reported skin itching (within the water-borne disease category). In the lower middle-class group, 48.3% (29) of participants reported not being affected, 35.7% (10) reported diarrhea, and 15.4% (2) reported skin itching. For the middle-class group, 11.7% (7) of participants reported not being affected, 3.6% (1) reported diarrhea, and none reported skin itching.

**Figure 5:**
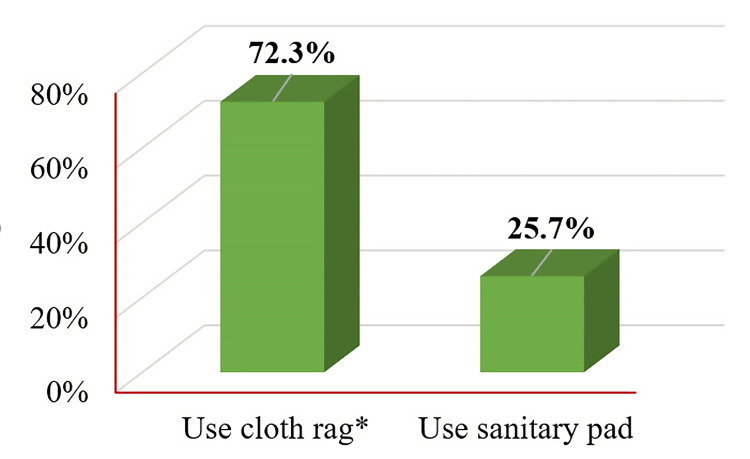
Distribution of menstrual hygiene practices among the study participants. * Reuse old cloth rags after washing and drying them in saline water.

Figure 5 displays the menstrual hygiene practices of the study participants. The majority, constituting 72.3% (73), reported using old cloth rags and reusing them after washing them in salt water. In contrast, 25.7% (26) reported using sanitary pads. Only 2.0% (2) indicated "N/A" (not applicable), which suggests either they did not provide a response or their menstrual hygiene practices were not captured by the given options.

## Discussion

Globally, seawater incursion is an insistent and persistent health concern at shoreline groundwater levels [17]. Hence, water bodies located on top of the land, like rivers, waterways/watercourses, canals, fishponds, etc., are ruthlessly ostentatious by the incursion of saline water [18]. Water bodies situated on top of the land and nearby surface drinking water of Bangladesh, India, and Vietnam, often called the Asian mega-delta coastal areas, are most liable to pollution by saline water invasion, enacting over 25 million people at difficulty of drinking saline water [18-20]. Global environmental change, which leads to a rise in sea level, increases the possibility of salinity of drinking water and promotes adverse health consequences, especially cardiovascular and hypertensive disorders [21-23]. The safe drinking water deficit is one of the most conspicuous coastal challenges [24,25]. Coastal populations around the globe primarily utilize saline water to meet their daily water needs, including drinking water in Bangladesh [26]. It has been reported that a considerable portion of the coastal population of Bangladesh is poverty-stricken [27,28]. Good menstrual hygiene management (MHM) execution varies internationally and depends on the person's social and financial status, individual inclinations, local customs, and dogmas. Adequate safe water supply and better quality and quantity of sanitation possessions lead to better MHM practices [29-31]. Women affected by poverty cannot maintain appropriate and adequate MHM practices [31,32].

A considerable portion of our study population utilizes pit latrines without water seals and drinks groundwater. Multiple studies revealed that after defecation, many pathogens decline after bowel evacuation. Nevertheless, these microbes frequently impair groundwater quality [33-38]. Islam et al. (2016) reported that "groundwater drawn from shallow tubewells in Bangladesh is often polluted by nearby pit latrines, which are commonly used toilets in rural and sub-urban areas of the country" [39]. ﻿It has been suggested that pit larine and drinking water sources should be at least 25 meters in "lateral distance" [39]. Misra and Paunikar (2023) reported that gastroenteritis develops because of drinking polluted water and unhygienic way of life [40]. Multiple studies reported that coastal populations, specifically low and middle-income countries (LMICs), develop diverse transmissible diseases, e.g., ophthalmic illnesses, gastroenteritis including cholera, skin infection, itch, and rashes. The reason behind these disorders is marginalized communities utilizing contaminated saline water for every house need including bathing and drinking [41-44]. This study subjects often suffer from diarrhea and itching skin, which is in the same line as earlier study reports. Friedrich-Ebert-Stiftung (FES), a German non-government organization, reported earlier that coastal female residents suffer from dysuria (painful urination), lower abdominal pain, fever, cough, etc. [45]. Our study subjects similarly suffer from painful urination and lower abdominal pain. Furthermore, the current study detected that the risk of diarrhea among tubewell water and rainwater consumers was significantly lower than among pond water users. Wu et al. (2011) revealed that improved access to tubewell water was related to lowering the possibility of diarrhea [46].

**Figure 6:**
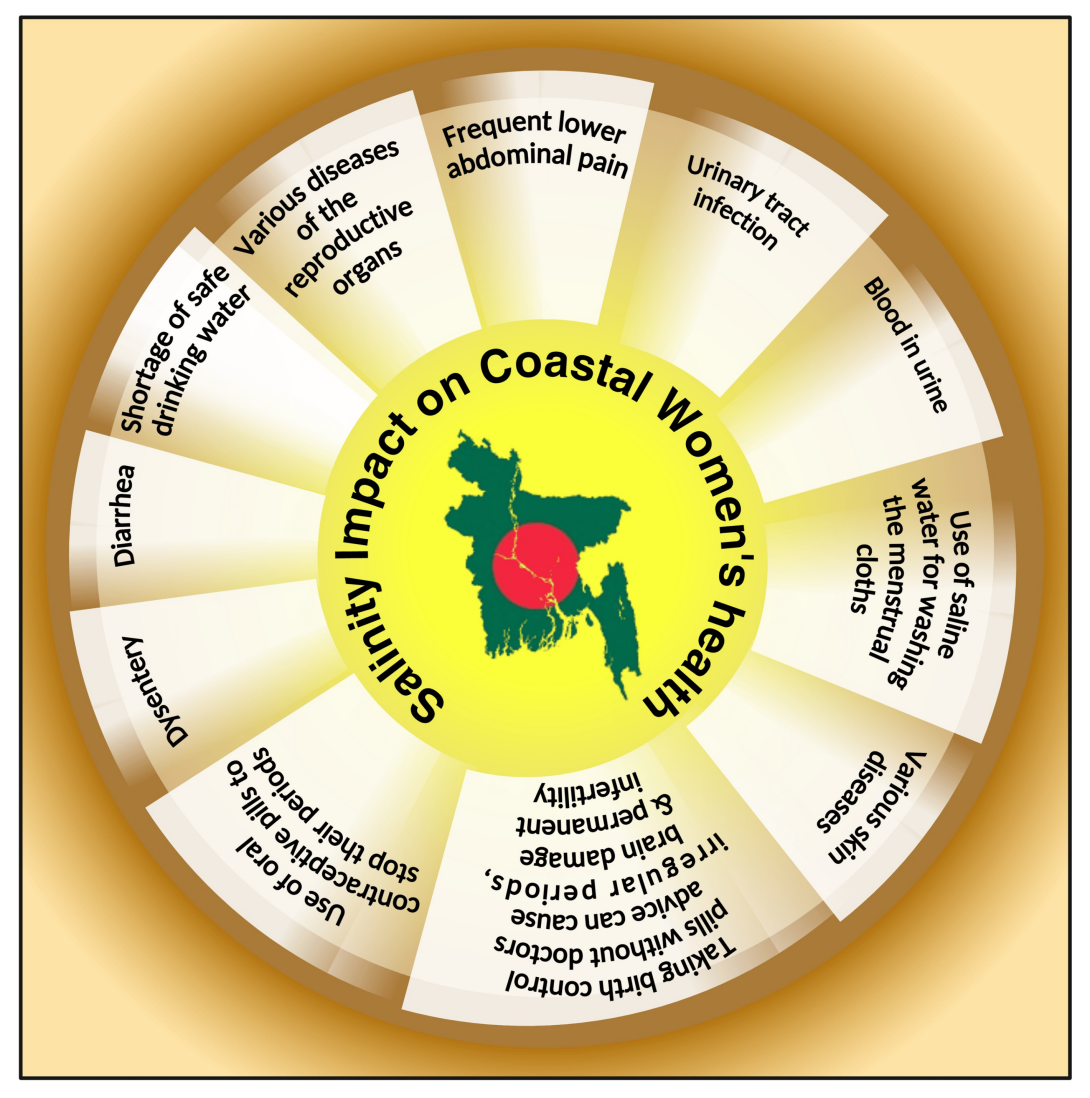
Illustration showing various detrimental health effects of saline water on coastal women's health. Note: This figure was drawn using the premium version of BioRender (https://biorender.com/) [16], with the agreement license number ZN272OZI5I. Image credit: Susmita Sinha.

Women are particularly susceptible as they have no alternatives except saline water during menstruation [29]. Over time, the reproductive health of women living along the coast becomes more vulnerable due to salinity intrusion (Figure 6) [29]. Women cope with the freshwater shortage by engaging in harmful behaviors without realizing the severe health-related adverse effects [47]. Ultimately, this causes long-term harm to their reproductive health [48]. People's health is in danger when unclean and contaminated water sources are available in the community. This may lead people to seek out other, riskier sources of water, which exposes them to illnesses, including cholera, typhoid, schistosomiasis, and diarrhea or dysentery [49]. The risk of diarrhea among tubewell water and rainwater users was shown to be considerably reduced in the current study by 0.25 times and 0.06 times, respectively, when compared to pond water users. The current study findings were similar to earlier published papers [50,51].

Limitations of the study

The study contained a small sample size and was confined to a limited area, which may have influenced the relevancy of the results to additional populations or geographical regions. Furthermore, the study used information that participants provided, which is prone to misinterpreting issues or evaluating them incorrectly.

## Conclusions

Disease outbreaks are caused mainly by saline intrusion from man-made ruins and events of nature that remove access to safe water for consumption. The government and non-governmental organizations should take action through an awareness campaign or by incorporating information about salinity and health into the curriculum to raise concern at the grassroots levels. This will help minimize the presence of water salinity in coastal areas of Bangladesh and improve people's access to standard health facilities. To provide a sustainable way of life for the current and future generations, it is also essential to encourage people to collect and store rainwater and desalinate water from nearby water sources.
